# Clinical and psychological characteristics of patients with ischemia and non-obstructive coronary arteries (INOCA) and obstructive coronary artery disease

**DOI:** 10.1016/j.ahjo.2023.100282

**Published:** 2023-02-23

**Authors:** Dinah L. van Schalkwijk, Jos Widdershoven, Michael Magro, Veerle Smaardijk, Maria Bekendam, Ilse Vermeltfoort, Paula Mommersteeg

**Affiliations:** a Center of Research on Psychology in Somatic Diseases (CoRPS), Department of Medical and Clinical Psychology, Tilburg University, the Netherlands; b Department of Cardiology, Elisabeth-TweeSteden Hospital, Tilburg, the Netherlands; c Department of Nuclear Medicine, Institute Verbeeten, Tilburg, the Netherlands

**Keywords:** Ischemia with non-obstructive coronary arteries (INOCA), Cardiovascular risk factors, Psychological distress, Well-being, Health-related quality of life, Medication use

## Abstract

**Study objective:**

Ischemia with non-obstructive coronary arteries (INOCA) is caused by vascular dysfunctions and predominantly seen in women. For better recognition and prevention more insight is needed on risk factors and well-being. We aimed to explore differences in psychological distress, quality of life, risk factors, and medication use between women with INOCA and obstructive coronary artery disease (CAD).

**Methods:**

Patients from two separate studies (*n* = 373, 57 % women) completed a questionnaire assessing psychological and clinical factors. Analyses were performed for women only who were categorized into three groups: non-ischemic chest pain (*n* = 115), INOCA (*n* = 68), and obstructive CAD (*n* = 30). Secondary analyses were performed for men only, and sex differences within INOCA patients were explored.

**Results and conclusion:**

Compared to obstructive CAD patients, INOCA patients reported better physical functioning (*p* = 0.041). Furthermore, INOCA patients had less often hypercholesterolemia (*p* < 0.001), were less often active smokers (*p* = 0.062), had a lower mean BMI (*p* = 0.022), and reported more often a familial history of CAD (*p* = 0.004). Patients with INOCA used antithrombotic, cholesterol lowering medications, and beta-blockers less often than patients with obstructive CAD. No differences between patients with INOCA and obstructive CAD were found for psychological distress, well-being, and for women-specific risk factors. The results suggest that women with INOCA experience similar levels of psychological distress and seem to have different risk factor profiles and are less optimally treated as compared to obstructive CAD patients. Further research on risk factors is needed for better prevention and treatment.

## 1 Introduction

An estimated 30–70 % of predominantly young, female patients with ischemic symptoms referred for coronary angiography (CAG) has ischemia with non-obstructive coronary arteries (INOCA) [1], [2], [3]. INOCA is a broad term covering different vascular dysfunctions contributing to abnormal coronary blood flow such as coronary microvascular dysfunction (CMD) and coronary vasospasm. In the present study INOCA is operationalized as having chest pain and detected ischemia in the absence of obstructive coronary artery disease [4]. A subgroup of the patients additionally received an index of microcirculatory resistance (IMR) measurement to detect CMD [5], however acetylcholine testing for coronary vasospasm could not be performed. INOCA is related to adverse cardiac outcomes [6], [7], recurrent angina symptoms, impaired quality of life (QoL), repeated hospitalizations [8], and psychological distress (e.g., depression, anxiety, stress) [9].

Psychological distress (e.g., depressive symptoms, anxiety, stress, and Type D personality) negatively affect the onset and prognosis of ischemic heart diseases and may be related to the etiology of INOCA [9], [10], [11]. It is unclear how psychological distress differs between patients with INOCA and patients with obstructive coronary artery disease (CAD). Both higher and similar levels of psychological distress in INOCA patients compared to obstructive CAD and non-cardiac patients have been found [12], [13], [14], [15]. The present study aims to further contribute to the controversy about psychological distress occurrences in INOCA patients.

Moreover, traditional cardiac risk factors (e.g., age, diabetes, hypertension, and obesity) are poorly related to the development of CMD, and only explain little variance in the occurrence of CMD [16], [17], [18]. Additionally, in a large cohort study the prevalence for diabetes was low and moderate for hypertension and hypercholesterolemia [18]. With regard to coronary vasospasm, smoking was found to be a risk factor whereas diabetes and hypertension were not [4]. Other emerging (e.g., inflammatory disorders, migraine) and women specific risk factors (e.g., pregnancy- and reproductive-related factors) have been associated with INOCA as well [19], [20], [21], [22]. Additional knowledge about risk factors is needed and could help earlier disease diagnosis and adjusting medical and non-medical interventions [4].

Treatment of INOCA is focused on reducing the effects of cardiac risk factors through lifestyle modifications and medication therapy (e.g., beta blockers, ACE/ARB inhibitors, statins, nitrates) [23]. Although this is important, previous research have shown that patients with INOCA less often use or continue cardiac preventive medication as compared to patients with obstructive CAD [7], [24].

This descriptive study investigates psychological distress, health-related QoL, well-being, traditional and emerging cardiac risk factors, and medication use in women with INOCA compared with women with obstructive CAD or non-ischemic chest pain. Identical analysis for men with INOCA and stratified analysis for sex differences in INOCA will also be performed and described as secondary results. The INOCA patients, irrespective of sex, are hypothesized to have a worse quality of life, worse well-being, more psychological distress, less traditional cardiac risk factors, a higher prevalence of emerging and (female) specific risk factors, and less cardiac medication use compared to obstructive CAD. Finally, we expect that women with INOCA have higher levels of psychological distress and poorer well-being compared to men.

## 2 Methods

### 2.1 Study design

#### 2.1.1 Participants and procedures

Data from two studies were combined and will be described respectively.

##### 2.1.1.1 The index of microcirculatory resistance (IMR) study

The IMR study was set up to examine cardiac, psychological, and sociodemographic factors related to CMD in symptomatic women suspected for INOCA. Patients were recruited between March 2019 and March 2020 from the Elisabeth-Tweesteden (ETZ) hospital Tilburg, the Netherlands. Patients were included when 1) experiencing recurrent cardiac symptoms without significant coronary obstructions; 2) additional coronary angiography with coronary function testing (but without acetylcholine spasm provocation tests) was offered for the detection of CMD; and 3) they had sufficient knowledge of the Dutch language.

Eligible patients (*N* = 134) received study information by e-mail, provided digital informed consent, and filled out an online questionnaire using Qualtrics (data processing agreement Tilburg University). Pen and paper questionnaires were available, which were sent and returned (coded) by postal mail. The questionnaires included demographic, clinical, women-specific, and psychological factors. Electronic medical records for cardiac risk factors, cardiac history, medication use, and diagnostic procedures were collected using the online Research Manager system for data collection (data processing agreement ETZ hospital).

**Fig. 1:**
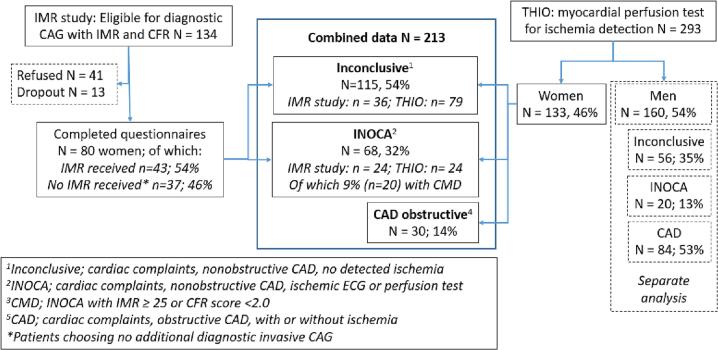
Flowchart of cardiac subgroups created from the IMR study and the THIO study. Subgroups were created based on presence (INOCA) or absence (Inconclusive; non-ischemic chest pain) of detected ischemia according to ECG (IMR study) or myocardial perfusion test (THIO study). In the INOCA group included via the IMR study, CMD was operationalized as having an IMR ≥ 25 or CFR ≤2.0. Obstructive CAD was presence of a history of obstructive CAD (<six months) either with or without current ischemia. CAD = obstructive coronary artery disease; CAG = coronary angiography; CMD = coronary microvascular dysfunction; IMR = index of microcirculatory resistance; IMR study = study in women eligible for additional diagnostic CAG with IMR and CFR measurement; INOCA = ischemia without obstructive coronary arteries; THIO = The Heart Inside Out study.

In total 80 (60 % response rate) provided written consent and completed the questionnaires. In total 43 (54 %) patients underwent an additional diagnostic CAG for coronary function testing, whereas 37 (46 %) patients chose to refrain from coronary function testing (Fig. 1 Flow Chart).

During invasive coronary function testing the Coronary flow reserve (CFR), index of microcirculatory resistance (IMR), and fractional flow reserve (FFR) were assessed [5]. CMD was defined as IMR ≥ 25 and/or CFR ≤2.0 [4], in the absence of current obstructive coronary artery disease within a six month time frame [4].

The study was approved by the hospital and Tilburg University (Medical) Ethics Committee (#L0.600.2018), and all patients provided written informed consent.

##### 2.1.1.2 The heart inside out (THIO) study

The THIO study was set up to examine emotions and psychological factors in patients referred for detection of myocardial ischemia. Patients were recruited between January 2017 and December 2018 at the Verbeeten Institute Tilburg, the Netherlands. The THIO study includes patients with cardiac complaints referred for a myocardial perfusion imaging single-photon emission computed tomography (MPI-SPECT; cardiac stress testing). The study has been described in more detail elsewhere [15].

In total 295 patients were included. Two respondents participated in both the THIO and the IMR study and were therefore only included in the IMR dataset. The 293 patients in the THIO study included both women (*N* = 133, 45 %) and men (*N* = 160, 55 %), who were examined separately (Fig. 1: Flow Chart).

Demographic and psychological factors were collected using a paper and pencil questionnaires. Information on cardiac risk factors, medication use, cardiac stress testing procedure, and medical history were retrieved from electronic medical records from the Elisabeth-Tweesteden hospital and the Verbeeten Institute.

Myocardial ischemia was detected semi-quantitatively by the nuclear physician using a summed difference score ≥ 2 as well as visual analysis, as was recommended by the American Society of Nuclear Cardiology [15]. Electronic medical records of CAG or computed tomography (CTCA) were examined for significant obstructions of the coronary arteries within a time frame of six months before or after the myocardial perfusion imaging.

### 2.2 Measures

#### 2.2.1 Groups according to cardiovascular measurement

Patients were grouped in line with the ‘diagnostic criteria for microvascular angina’ as described by the ESC consensus paper [4]. Study equivalent steps are summarized in Supplemental Table S1 and depicted in the Flowchart (Fig. 1). In total three subgroups (INOCA, obstructive CAD, non-ischemic chest pain (inconclusive)) were created based on: (1) presence of cardiac symptoms (e.g., chest pain, angina, and/or dyspnea), (2) presence/absence of obstructive CAD in the past six months based on most recent CAG or coronary computed tomographic angiography, (3) presence/absence of ischemia detected by ECG changes during an episode of chest pain in the emergency room or most recent stress testing (in IMR study) or detected by myocardial perfusion imaging (in THIO study),(4) presence of impaired microvascular functioning detected by an abnormal coronary function test (IMR ≥ 25 and/or CFR ≤2.0) (Table S1). Patients were classified as non-ischemic chest pain (inconclusive) if they had cardiac symptoms, but additional diagnostic examination did not show the presence of ischemia. It should be noted that in the non-ischemic chest pain group, other cardiac causes, such as coronary vasomotor dysfunction (e.g., vasospasm), could not be excluded.

#### 2.2.2 Traditional and emerging risk factors

Sociodemographic, traditional, and women-specific risk factors, comorbid conditions, and medication use were obtained from questionnaires and hospital records. For descriptive purposes, variables were dichotomized when multiple categories were present. Medication use was grouped according to corresponding Anatomical Therapeutic Chemical (ATC) codes [25].

#### 2.2.3 Psychological distress, well-being, and quality of life

Validated questionnaires were used to examine psychological distress (depression, anxiety, perceived stress, and Type D personality), health-related QoL, well-being and fatigue. Health-related quality of life (HRQoL) was assessed with the modified Seattle Angina Questionnaire (modified-SAQ-7) containing seven items on cardiac symptoms [26]. Higher scores indicate better HRQoL. Patients reporting having had any chest pain in the past month according to the modified SAQ were dichotomized as ‘chest pain’ (versus none).

The Mental Health Continuum-Short Form (MHC-SF) measures well-being of patients [27]. The MHC-SF consists of 14 items and assesses the averaged three components of well-being: emotional (3 items), psychological (6 items) and social (5 items), as well as an (averaged) total score for well-being. A higher score indicates better well-being.

The Fatigue Assessment scale (FAS-10) was used to assess fatigue and consists of the sum of ten questions reflecting mental and physical fatigue on a 1–5 scale [28].

Depressive symptoms were assessed with the Dutch version of the Patient Health Questionnaire (PHQ-9) [29]. This nine-item self-report instrument measures the presence and severity of depressive symptoms on a 0–3 scale. Patients were asked to indicate the frequency of several depressive symptoms during the last two weeks. A cut-off score of 10 or higher was used to define moderate or severe depressive symptoms.

Anxiety symptoms were assessed with the 7-item General Anxiety Disorder questionnaire (GAD-7) [30]. Patients were asked how often they have suffered from each of the seven core symptoms of generalized anxiety disorder, during the last two weeks, on a 0–3 scale. A cut-off score of 10 or higher was used to define moderate or severe symptoms of anxiety.

Stress perception was measured with the Perceived Stress Scale (PSS) [31]. This ten-item instrument assesses on a 0–4 scale, to which degree patients appraise situations in one's life as stressful. Patients are asked to indicate how unpredictable, uncontrollable, and overloaded their lives have been during the previous month. A total sum score was calculated, measuring perceived stress, with a higher score indicating more stress.

Type D personality (a combination of social inhibition (SI) and negative affectivity (NA)) was assessed with the 14-item Type D scale (DS14) [32]. In addition to using the continuous scores for SI and NA, a cut-off of 10 on both subscales was used to identify Type D personality.

### 2.3 Statistical analysis

Findings were reported for women grouped by non-ischemic chest pain (inconclusive), INOCA, and obstructive CAD. Group differences were examined using Pearson's Chi-squared test for categorical variables and One-way ANOVA for continuous variables. When main group differences were present, additional Chi-squared tests were conducted with post-hoc Bonferroni adjusted z-tests for independent proportions to examine which groups differed from one another. Post-hoc comparisons for significant group differences in the One-way ANOVA were examined using least significant difference (LSD). The primary analysis was performed in women only since INOCA is more prevalent in women. Secondary analyses examined differences between groups for men, and between women with INOCA versus men with INOCA using One-way ANOVA or Chi-squared tests. Secondary results will be reported in a supplemental file. Analyses were conducted using IBM SPSS Statistics for windows, version 27 [33].

## 3 Results

### 3.1 Patients' characteristics stratified by cardiac patient group

Table 1 shows the descriptive and medical characteristics of women stratified by cardiac group. In total 213 women were included, of whom 54 % (*n* = 115) were classified as inconclusive (non-ischemic chest pain), 32 % (*n* = 68) as INOCA, and 14 % (*n* = 30) as obstructive CAD. The mean age was 63.3 (±9.2) years. Post-Hoc tests showed that women with Patients with INOCA reported more often being with a partner (90 % vs. 63 %) and were more often higher educated (26 % vs. 3 %) compared to patients with obstructive CAD.

**Table 1:** Patients' characteristics of women, stratified by cardiac groups.

	Inconclusive		INOCA		Obstructive CAD		Test-value	p-Value
(*N* = 213)	54 %	115	32 %	68	14 %	30		
Age [years]	63.03	9.16	62.74	9.54	65.47	8.62	1.00	0.367

Sociodemographic factors								
Having a partner	73 %^a^	83	90 %^a^	60	63 %^a^	19	10.18	0.006
College education or higher	21 %	24	26 %^a^	17	3 %^a^	1	6.80	0.033
Paid work [versus other]	33 %	38	41 %	27	20 %	6	4.06	0.132

Lifestyle factors								
BMI [kg/m2]	27.72	5.88	27.04^a^	4.93	30.76^a^	8.68	3.90	0.022
Obesity [BMI ≥30]	25 %	28	25 %	16	45 %	13	4.97	0.083
Current smoker	11 %	12	3 %^a^	2	17 %^a^	5	5.55	0.062
Any alcohol use	53 %	60	51 %	34	59 %	17	0.50	0.777
Being physically active	67 %	75	69 %	46	62 %	18	0.40	0.819

Cardiovascular risk factors								
Family history of heart disease	50 %	57	70 %^a^	47	37 %^a^	11	11.22	0.004
Hypertension	56 %	58	73 %	48	46 %	11	7.25	0.027
Hypercholesterolemia	50 %	58	65 %^a^	44	93 %^a^	28	18.98	<0.001
Diabetes	28 %	32	18 %	12	30 %	9	2.86	0.240

Women-specific factors								
Age of onset menarche [years]	13.20	1.78	14.01	5.04	13.39	1.73	1.33	0.267
Pregnancy	96 %	109	94 %	63	90 %	27	1.41	0.494
Pregnancy complications	44 %	48	37 %	23	38 %	10	1.12	0.571
Ovary or uterus extirpation	26 %	30	30 %	20	52 %	15	7.04	0.030
Menopausal	94 %	105	88 %	60	90 %	26	1.76	0.414
Vasomotor symptoms experienced in the past week: sweat attack or hot flashes	59 %	67	61 %	41	53 %	16	0.53	0.768
Comorbid conditions								
Migraine	24 %	28	22 %	15	23 %	7	0.13	0.939
Fibromyalgia	13 %	15	13 %	9	7 %	2	1.00	0.606
Thyroid condition	19 %	22	15 %	10	10 %	3	1.66	0.436
Chronic Fatigue Syndrome	11 %	12	16 %	10	14 %	4	0.69	0.710
Allergic condition	28 %	29	23 %	15	29 %	8	0.44	0.804
Chronic pain	45 %	48	36 %	23	26 %	7	3.90	0.142

Medication use [ATC code]								
Cardiac medication								
Antithrombotics [B01A]	43 %	50	59 %^a^	40	93 %^a^	28	24.4	<0.001
Cholesterol lowering [C10A]	35 %	40	49%^a^	33	93 %^a^	28	32.8	<0.001
Diuretics [C03]	20 %	23	22 %	15	23 %	7	0.21	0.900
ACE/ARB inhibitors [C09]	32 %	37	28 %	19	50 %	15	4.71	0.095
Beta blockers [C07]	32 %	37	26 %^a^	18	67 %^a^	20	15.6	<0.001
Calcium inhibitor [C08]	28 %^a^	32	50 %^a^	34	20 %^a^	6	12.4	0.002
Nitrates [C01DA/DX]	23 %	27	38 %	26	50 %	15	9.53	0.009
Other medication								
Antidepressants [N06AB/AA/AX]	15 %	17	15 %	10	7 %	2	1.43	0.488
Benzodiazepines [N05B/N05C]	13 %	15	13 %	9	10 %	3	0.23	0.893
COPD medication [A07/R03]	17 %	20	10 %	7	10 %	3	2.26	0.323
Diabetes medication [A10A/A10B]	13 %	15	10 %	7	23 %	7	3.08	0.215
Hormone Replacement [G03/L02]	2 %	2	3 %	2	7 %	2	2.12	0.347
Gastric medication [A02]	43 %	49	41 %	28	53 %	16	1.36	0.506
Thyroid medication [H03AA01]	17 %	20	12 %	8	7 %	2	2.71	0.258

*Note*: Percentage and N or mean and SD are reported. Test values are Chi-squared or F-value.

Inconclusive group has non-ischemic chest pain; cardiac symptoms, without detected ischemia in the absence of obstructive CAD.

a INOCA group is significantly different from the group(s) with ^a^ using a post-hoc Bonferroni corrected z-test.

#### 3.1.1 Risk factors and comorbid conditions

Patients with INOCA more often reported a family history of heart diseases (70 %) as compared to patients with obstructive CAD (37 %).The Pearson Chi-Squared test was significant for hypertension, however, pair wise comparison test showed no further group differences. Hypercholesterolemia was less often reported in the INOCA group as compared to the obstructive CAD group (65 % vs. 93 %). Moreover, patients with INOCA were less often current smokers and had a significant lower mean BMI. No significant group differences were found for, hypertension, diabetes, women-specific risk factors, nor for the comorbid conditions migraine, fibromyalgia, thyroid condition, chronic fatigue syndrome, allergic conditions, and chronic pain (Table 1).

#### 3.1.2 Medication use

Regarding medication use, patients with INOCA less frequently used antithrombotics, cholesterol lowering medications, and beta blockers as compared to the obstructive CAD group. In contrast, the INOCA group more often had calcium inhibitors as compared to the obstructive CAD and inconclusive group.

#### 3.1.3 Quality of life, well-being, and psychological distress

Table 2 shows group findings for health-related quality of life, well-being, and psychological distress. Women with INOCA reported higher physical functioning according to the modified SAQ compared to women with obstructive CAD. No differences were found for psychological distress, well-being, fatigue, angina frequency, and chest pain presence.

**Table 2:** Health status, well-being, and psychological distress in women, stratified by cardiac group.

	Inconclusive		INOCA		CAD obstructive		Test-value	p-Value
Health status and well-being								
Modified SAQ								
Chest pain past month	79 %	91	73 %	49	67 %	20	2.29	0.319
Physical limitation	53.33	22.07	60.00^a^	19.84	50.00^a^	21.96	2.77	0.065
Angina frequency	77.66	17.58	80.76	18.75	77.59	20.81	0.64	0.528
Quality of life	62.84	25.08	64.81	26.51	56.03	27.06	1.18	0.310
Mental health continuum								
Emotional wellbeing	3.45	0.91	3.54	1.03	3.44	1.21	0.18	0.833
Social wellbeing	2.60	0.93	2.60	1.08	2.61	1.11	0.00	0.999
Psychological wellbeing	3.25	1.03	3.29	1.07	3.15	1.07	0.17	0.841
Wellbeing total	3.06	0.83	3.10	0.92	3.02	1.00	0.08	0.919
Fatigue [FAS10]	24.16	7.29	24.38	7.06	25.54	6.96	0.44	0.645

Psychological distress								
Depressive symptoms [PHQ9]	6.02	5.38	6.20	5.31	5.68	4.35	0.10	0.906
Moderate depression [≥10]	20 %	22	21 %	14	17 %	5	0.20	0.903
Anxiety [GAD7]	5.64	5.08	6.01	5.56	4.86	4.47	0.51	0.604
Moderate anxiety [≥10]	18 %	20	29 %	19	10 %	3	5.27	0.072
Perceived stress [PSS]	15.72	6.85	15.80	7.61	15.76	7.36	0.00	0.997
Negative affectivity [DS14NA]	9.86	5.76	11.06	5.78	9.14	4.33	1.49	0.229
Social inhibition [DS14SI]	7.61	5.74	7.52	4.86	7.93	6.13	0.05	0.947
Type D personality	21 %	24	28 %	19	25 %	7	1.11	0.574

*Note*: Percentage and N or mean and SD are reported. Test values are Chi-squared or F-value.

One-way ANOVA for the quality of life subscale physical limitation showed that group means did not differ, but post-hoc test did show a significant difference between the INOCA and CAD obstructive group (*p* = 0.041).

a INOCA group is significantly different from the group(s) with ^a^ using a post-hoc Bonferroni corrected z-test.

### 3.2 Clinical characteristics for the coronary function test

Table 3 presents the results of the coronary function tests using the index of microcirculatory resistance and CFR. These results are only available for women who participated in the IMR study. Mean values for IMR, CFR, and FFR were: 20.89 ± 10.92, 3.04 ± 1.42, 0.93 ± 0.05, respectively. In total 47 % (*n* = 20/43) of the tested patients had CMD according to either an IMR ≥ 25 or a CFR ≤ 2, of which 65 % (13/20) had an IMR ≥ 25, and 55 % (11/20) had a CFR ≤ 2. In total nine patients (45 %, 9/20) had an IMR ≥ 25 but with a CFR > 2, seven patients (35 %, 7/20) had a CFR ≤ 2 but with an IMR < 25, and only four patients (20 %, 4/20) had both an IMR ≥ 25 and a CFR ≤ 2. The remaining 23 patients (53 %, 23/43) did not meet either CMD criteria according to the IMR measurement and had both an IMR < 25 and a CFR > 2.

**Table 3:** Coronary function testing measures in CMD.

	Inconclusive		INOCA		CMD		Test-value	p-Value
	% or mean	N or SD	% or mean	N or SD	% or mean	N or SD		
*N* = 43	37 %	16	16 %	7	47 %	20		
Mean IMR-value	15.63	4.19	13.80	6.13	27.59	12.21	10.19	<0.001
IMR < 25	100 %	16	100 %	7	35 %	7	21.43	<0.001
IMR ≥ 25 [CMD]	0 %	0	0 %	0	65 %	13		
Mean CFR-value	3.86	1.25	0.71	4.07	1.25	2.06	15.84	<0.001
CFR > 2	100 %	15	100 %	7	45 %	9	16.39	<0.001
CFR ≤ 2 [CMD]	0 %	0	0 %	0	55 %	11		
Mean FFR-value	0.92	0.96	0.86	0.96	0.96	0.91	1.49	0.238

*Note*: This table demonstrates the clinical test results of the coronary function test whereby the index of microcirculatory resistance was used. This table only includes participants from the IMR-study who underwent invasive ‘IMR’ testing. The INOCA subgroup CMD was defined as having an IMR ≥25 or a CFR ≤ 2. Percentage and N or mean and standard deviation are reported. Test values are Chi-squared or F-value. IMR = index of microcirculatory resistance, CFR = coronary flow reserve, INOCA – ischemia with non-obstructive coronary arteries, CMD = coronary microvascular disease.

### 3.3 Secondary analysis of men, and sex differences

Results of the secondary analysis are reported in the supplemental file (Supplemental tables S2, S3, S4, S5). Men with INOCA more often reported comorbid chronic fatigue syndrome (17 %) as compared to the obstructive CAD group (0 %) and less often used antithrombotics (80 % vs. 96 %). No other significant differences, including health-related quality of life, well-being, psychological distress, and health status (comorbidities and medication use) were observed. Women with INOCA have a higher prevalence of cardiac family history and hypertension, but are less often smokers, less often used any alcohol, less often reported diabetes, and less often used ACE/ARB, beta blockers, and diabetes medication compared to men with INOCA.

## 4 Discussion

We aimed to describe psychological and clinical characteristics of patients with INOCA in comparison with obstructive CAD and non-cardiac patients. Our findings showed that patients with INOCA did not experience more psychological distress and did not have a worsen well-being as compared to patients with obstructive CAD. Surprisingly, patients with INOCA reported better physical functioning. Furthermore, in the INOCA group hypercholesterolemia was less often reported, while a family history of cardiac disease was more often reported in the INOCA group. No differences were found for women-specific risk factors. Lastly, in line with our expectations, INOCA patients did use certain medication less often than patients with obstructive CAD (antithrombotic, cholesterol lowering medication, and beta blockers). Sex stratified analysis within the INOCA group showed that women have a higher prevalence of cardiac family history and hypertension, but less often reported smoking, using alcohol, having diabetes, use ACE/ARB, beta blockers, or diabetes medication compared to men.

Contrary to our expectations, patients with INOCA experienced similar levels of psychological burden (depression, anxiety, stress, and Type-D personality) and well-being as compared to obstructive CAD patients. In older studies it was found that patients with ‘syndrome X' (older term to describe symptomatic patients with non-obstructive coronary arteries) had higher levels of psychological morbidity [12], [34]. However, conclusions in more recent studies are in line with our results whereby no differences in psychological distress were found except for cardiac anxiety [14], [15], [35]. These findings suggest that cardiac symptoms (e.g., chest pain) leads to psychological distress, regardless of the cardiac disease type or disease severity [36], [37], [38].

Although it is likely that INOCA patients do not experience higher levels of psychological burden as compared to obstructive CAD patients, it seems that psychological comorbidities such as depression, anxiety, and stress play a prominent role in the development and prognosis of INOCA. In addition, younger women with ischemic heart diseases seem to be more vulnerable to psychological stress risk factors. For instance, mental stress-induced myocardial ischemia is more common in women [39]. It is suggested that psychological stress through dysfunctional vasoreactivity, autonomic nervous system, and/or increased inflammation may be involved in the etiology of INOCA [9], [39], [40], [41]. Therefore, future research should focus on further understanding these mechanisms, psychological stress reducing interventions, and the role of screening for psychological risk factors in INOCA patients.

Moreover, in the current study, patients with INOCA showed better physical functioning and similar levels of QoL as compared to patients with obstructive CAD. This contradicts with previous studies, where lower physical functioning and QoL were observed [14], [42]. A possible explanation for the results may be the timing of questionnaire completion. In the THIO study, the questionnaire was administered during the diagnostic examination (before diagnosis), whereas in the IMR study it was administered after the diagnostic examination. Thus, patients were aware of their diagnosis and were treated for their symptoms. The combination of a definitive diagnosis and appropriate treatment may have improved their QoL and physical functioning, as supported by existing literature [43], [44], [45]. More insight is needed into how QoL may vary within different moments of the health care trajectory.

The present study found differences in traditional risk factors for the INOCA group compared with the obstructive CAD group. A history of familial heart disease and hypercholesterolemia were more common in patients with INOCA compared with the obstructive CAD patients. Also, women with INOCA reported less often being active smokers and on average had a lower mean BMI than obstructive CAD patients. Differences in smoking prevalence were also found by Konst et al. [14], but for other traditional risk factors, they found no significant differences between INOCA and CAD patients. Interestingly, other studies have observed higher prevalence's of hypertension and dyslipidaemia and lower prevalence's of active smokers and diabetes in INOCA patients. Our and previous results indicate different risk factor profiles and estimation that should be considered in patients with INOCA [18], [46]. However, a clearer understanding of the risk profiles in INOCA patients is still needed. Therefore, future research with larger samples should provide more insight into the prevalence of risk factors and the underlying mechanisms. In this regard, it is important to distinguish between the two endotypes (vasospasm and CMD), as the findings of the CorMicA trial indicate disparities in risk factors between the two [42].

In line with previous findings by Konst et al., no differences were found for women-specific risk factors, between patients with INOCA and obstructive CAD [14]. Furthermore, in another study no significant associations between a history of reproductive risk factors and a proxy measure for CMD were found [47]. This is contradictory since there is evidence for female-specific risk factors to be associated with a higher risk of cardiovascular diseases [46]. The inconsistency within the literature warrants for more clinical trials researching women-specific risk factors in relation to the development of INOCA.

Risk factor management is important in patients with INOCA because they have an increased risk of adverse events (e.g. myocardial infarction, death) and an impaired health status [8], [48]. Therefore, medication treatment for risks reduction seems to be crucial. In the present study it was found that both patients with INOCA less often used antithrombotics, cholesterol lowering medication, and beta blockers. Similar findings were found for beta blockers and cholesterol lowering medication in another study [42].

A limited number of studies have investigated differences in medication use between INOCA and obstructive CAD. The current results and other studies support that patients with INOCA are more often undertreated [24], [42]. However, medication therapy seems to have beneficial effects on symptom reduction and quality of life [45], [48]. Upcoming large trials, such as the WARRIOR trial are promising and important for further insights on the effects of cardioprotective medications on the different INOCA endotypes (CMD and vasospasm) [49]. A possible bias in medication prescription to men and women may play a role [50], [51]. A study by Mommersteeg et al. [25] examined medication use in suspected INOCA patients stratified for sex. In this study no differences were found. This is contradictory to our findings showing that women less often reported the use of ACE/ARB inhibitors and beta blockers as compared to men with INOCA. A meta-analysis evaluating sex differences in cardiovascular medication prescription showed that ACE inhibitors and beta-blockers (only among patients with established cardiovascular disease) were less likely prescribed to women [52]. However, a previous study found that ACE-I treatment is important in patients with signs and symptoms of INOCA, as ACE-I significantly improved CFR and reduced angina frequency [53]. This was not found for adjuvant treatment with ARB [54]. A second explanation for undertreatment is discontinuation of medication which was found to be more prevalent in patients with INOCA [24]. However, this is only a speculation as this was not measured in the current study.

### 4.1 Limitations

Since the present study is explorative and consists of small group numbers, the results should be interpreted with caution and should not be generalized to all INOCA patients. As stated above, data sets of two different studies were combined. Hence, the moment of questionnaire completion differed. This may have influenced significant and null findings. Patients from the IMR-study underwent coronary function testing, albeit at the time without spasm provocation testing with Acetylcholine, for the diagnosis of CMD. Therefore, no distinction could be made between patients with CMD and vasospasm or both. Furthermore, because most participants did not undergo coronary function testing, a definitive diagnosis of INOCA could not be made in most participants. Therefore, participants were suspected of INOCA based on diagnostic criteria. Finally, the shortcoming of the SPECT scan should also be mentioned, as this imaging method is less sensitive due to low-resolution images and is unable to measure coronary flow reserve (CFR).

## 5 Conclusion

This cross-sectional explorative study suggests that patients with INOCA experience similar levels of psychological distress and well-being as compared to patients with obstructive CAD. Against expectations, patients with INOCA reported better physical functioning. Because of the prominent role of psychological distress in the onset and prognosis of INOCA we advocate for further research on the bidirectional mechanism and the effect of targeted intervention. The data also show slight differences in risk factors that may indicate differences in risk profiles. Finally, we concluded that INOCA patients seem to be less optimally treated, which is worrisome given the fact that a targeted medication treatment is beneficial to symptoms and quality of life. Larger RCT studies should be performed to better understand which risk factors influence the diseases and whether these could help to recognize INOCA endotypes in an earlier state.

## Funding

This work was supported by ‘We Care’ from 10.13039/501100007659Tilburg University and Elisabeth-Tweesteden hospital [grant number WeCare2019] and the 10.13039/501100002996Dutch Heart Foundation (de Hartstichting) [grant number #2019T102].

## CRediT authorship contribution statement

The authors confirm contribution to the paper as follows: Conception and design of the study: D. L. van Schalkwijk, P. M. C. Mommersteeg, J. W. M. G. Widdershoven; Data collection: D. L. van Schalkwijk, J. W. M. G. Widdershoven, V. Smaardijk, M. Bekendam, I. Vermeltfoort, P. M. C. Mommersteeg; analysis and interpretation of results: D. L. van Schalkwijk, P. M. C. Mommersteeg; Draft manuscript preparation: D. L. van Schalkwijk, J. W. M. G. Widdershoven, P. M. C. Mommersteeg; Critical revision of the article: all authors; Final approval of the version to be published: all Authors.

## Declaration of competing interest

The authors declare that they have no known competing financial interests or personal relationships that could have appeared to influence the work reported in this paper.

## Data Availability

The data underlying this article will be shared on reasonable request to the corresponding author.

## Appendix A Supplementary data

Supplementary material Image 1
