# Supplementary material for: Clinical and psychological characteristics of patients with ischemia and non-obstructive coronary arteries (INOCA) and obstructive coronary artery disease
Source: Am Heart J Plus. 2023 Feb 23;27:100282. doi: 10.1016/j.ahjo.2023.100282 (PMC10945986; doi:10.1016/j.ahjo.2023.100282)
Supplement: Supplementary file 1 — Supplementary material [file mmc1.docx]

**Clinical and Psychological characteristics of patients with ischemia and non-obstructive coronary arteries (INOCA) and obstructive coronary artery disease.**

Dinah van Schalkwijk MSc, Jos Widdershoven MD PhD, Michael Magro MD PhD, Veerle Smaardijk PhD, Maria Bekendam MSc, Ilse Vermeltfoort MD PhD, Paula Mommersteeg PhD.

**SUPPLEMENTARY MATERIAL**

**Contents:**

Supplementary Table S1 - Use of diagnostic criteria for microvascular angina and equivalent in the present study

Results secondary analysis for men stratified by cardiac group

Supplementary Table S2 - Patient characteristics of men, stratified by cardiac groups

Supplementary table S3 - Health status, well-being, and psychological distress in men, stratified by cardiac groups

Results secondary analysis for INOCA patients stratified for sex

Supplemental Table S4. INOCA patients’ characteristics stratified by sex

Supplemental table S5 INOCA patients’ health status, well-being, and psychological distress, stratified by sex

## Supplemental Table S1. Use of diagnostic criteria for microvascular angina and equivalent in the present study. Adapted after Kunadian Table 1 ^[[1]](#footnote-1)^

| **Criterium** | **Evidence** | **Diagnostic parameter** | **Equivalent in present study** | **Group in present study** |
| --- | --- | --- | --- | --- |
| 1 | Symptoms of myocardial ischemia | Effort or rest angina  Exertional dyspnea | Electronic medical records with reason for referral in THIO and IMR study | **Inconclusive**: cardiac symptoms, no evidence of ischemia; no history of obstructive CAD |
| 2 | Absence of obstructive CAD | <50% diameter reduction or FFR >0.80 based on:   - Coronary CTA - Invasive coronary angiography | Electronic medical records for most recent CAG or CTA, examined conclusion and date.  Cardiac history of myocardial infarction, percutaneous coronary intervention, and coronary artery bypass graft surgery. | **CAD obstructive**: cardiac symptoms, presence of obstructive history either with or without evidence of ischemia |
| 3 | Objective evidence of myocardial ischemia | Presence of reversible defect, abnormality or flow reserve on a functional imaging test. | IMR study: ischemic ECG during an episode of chest pain while in the emergency room, or ischemic ECG during most recent stress testing  THIO study: ischemia as detected by myocardial perfusion imaging | **INOCA**: cardiac symptoms, evidence of ischemia according to ECG or myocardial perfusion imaging, non-obstructive CAD, or obstructive > 6 months ago |
| 4 | Evidence of impaired coronary microvascular function | - Impaired coronary flow reserve (cut-off <2.0), invasive or noninvasively determined - Abnormal coronary microvascular resistance indices (e.g., IMR >_25) - Coronary microvascular spasm, defined as reproduction of symptoms, ischemic ECG shifts but no epicardial spasm during acetylcholine testing | IMR study: impaired coronary microvascular function with either CFR <2.0 or IMR ≥ 25. | **CMD**:  Same as INOCA, but with IMR ≥ 25 or CFR score <2.0  Note: coronary vasospasm was not measured |

*Note:* CAD= coronary artery disease; CTA= computed tomographic angiography; ECG= electrocardiogram; ER= emergency room; MIBI-SPECT= myocardial perfusion imaging single-photon emission computed tomography FFR=fractional flow reserve; IMR= index of microcirculatory resistance.

## Results secondary analysis for men stratified by cardiac group

Results for men stratified by cardiac group can be found in Supplemental Table S2 and Supplemental Table S3. The distribution of men per group was 35%, 13%, and 53% for inconclusive patients, INOCA, and obstructive CAD respectively. A family history of heart disease was less often present in men with INOCA (17%) as compared to men with obstructive CAD (49%). Men with INOCA more often reported comorbid chronic fatigue syndrome (17%) as compared to the obstructive CAD group (0%) and less often used antithrombotics (80% vs. 96%). No other significant differences, including health-related quality of life, well-being, psychological distress, and health status (comorbidities and medication use) were observed.

## Supplemental Table S2. Patient characteristics of men, stratified by cardiac groups

|  | Inconclusive | | INOCA | | CAD obstructive | | Test-value | p-value |
| --- | --- | --- | --- | --- | --- | --- | --- | --- |
| Group prevalence (N=160) | 35% | 56 | 13% | 20 | 53% | 84 |  |  |
| Age [years] | 68.55 | 6.85 | 66.30 | 11.98 | 67.51 | 9.13 | 0.53 | 0.588 |
| ***Sociodemographic factors*** |  |  |  |  |  |  |  |  |
| Having a partner | 93% | 52 | 89% | 17 | 94% | 78 | 0.49 | 0.783 |
| College education or higher | 32% | 18 | 32% | 6 | 18% | 15 | 4.28 | 0.117 |
| Paid work [versus other] | 21% | 12 | 21% | 4 | 25% | 21 | 0.30 | 0.861 |
| ***Lifestyle factors*** |  |  |  |  |  |  |  |  |
| BMI [kg/m2] | 27.62 | 4.97 | 29.05 | 2.86 | 27.96 | 4.49 | 0.67 | 0.512 |
| Obesity [BMI>=30] | 31% | 16 | 39% | 7 | 26% | 21 | 1.30 | 0.522 |
| Current Smoker | 11% | 6 | 25% | 5 | 18% | 15 | 2.55 | 0.280 |
| Any alcohol use | 66% | 37 | 79% | 15 | 70% | 57 | 1.14 | 0.566 |
| Being physically active | 63% | 35 | 61% | 11 | 60% | 50 | 0.07 | 0.965 |
| ***Cardiovascular risk factors*** |  |  |  |  |  |  |  |  |
| Family history of heart disease | 27% | 15 | **17%^a^** | **3** | **49%^a^** | 41 | 10.36 | 0.006 |
| Hypertension | 50% | 25 | 47% | 9 | 51% | 38 | 0.10 | 0.951 |
| Hypercholesterolemia | 55% | 31 | 75% | 15 | 88% | 74 | 19.21 | <0.001 |
| Diabetes | 32% | 18 | 45% | 9 | 39% | 33 | 1.28 | 0.527 |
| ***Comorbid conditions*** |  |  |  |  |  |  |  |  |
| Migraine | 5% | 3 | 15% | 3 | 7% | 6 | 2.01 | 0.366 |
| Fibromyalgia | 2% | 1 | 0% | 0 | 0% | 0 | 1.87 | 0.393 |
| Thyroid condition | 4% | 2 | 10% | 2 | 2% | 2 | 2.61 | 0.272 |
| Chronic Fatigue Syndrome | 2% | 1 | **17%^a^** | **3** | **0%^a^** | **0** | 15.03 | 0.001 |
| Allergic condition | 28% | 14 | 17% | 3 | 13% | 10 | 4.42 | 0.110 |
| Chronic Pain | 26% | 13 | 39% | 7 | 17% | 13 | 4.17 | 0.124 |
| ***Medication use*** |  |  |  |  |  |  |  |  |
| *Cardiac medication* |  |  |  |  |  |  |  |  |
| Antithrombotics [B01A] | 54% | 30 | **80%^a^** | **16** | **96%^a^** | **81** | 37.70 | <0.001 |
| Cholesterol lowering [C10A] | 46% | 26 | 70% | 14 | 87% | 73 | 26.54 | <0.001 |
| Diuretics [C03] | 18% | 10 | 10% | 2 | 26% | 22 | 3.12 | 0.210 |
| ACE/ARB inhibitors [C09] | 46% | 26 | 55% | 11 | 63% | 53 | 3.81 | 0.149 |
| Beta blockers [C07] | **46%^a^** | **26** | **80%^a^** | **16** | 60% | 50 | 7.09 | 0.029 |
| Calcium inhibitor [C08] | 20% | 11 | 35% | 7 | 42% | 35 | 7.39 | 0.025 |
| Nitrates [C01DA/DX] | **13%^a^** | **7** | **40%^a^** | **8** | 51% | 43 | 21.90 | <0.001 |
| *Other medication* |  |  |  |  |  |  |  |  |
| Antidepressants [N06AB/AA/AX] | 4% | 2 | 10% | 2 | 5% | 4 | 1.30 | 0.521 |
| Benzodiazepines [N05B/N05C] | 7% | 4 | 5% | 1 | 2% | 2 | 1.84 | 0.398 |
| COPD medication [A07/ R03] | 11% | 6 | 5% | 1 | 12% | 10 | 0.81 | 0.666 |
| Diabetes medication [A10A/ A10B] | 11% | 6 | 30% | 6 | 23% | 19 | 4.70 | 0.095 |
| Hormone Replacement [G03/ L02] | 2% | 1 | 0% | 0 | 0% | 0 | 1.87 | 0.393 |
| Gastric medication [A02] | 46% | 26 | 50% | 10 | 63% | 53 | 4.07 | 0.130 |
| Thyroid medication [H03AA01] | 4% | 2 | 5% | 1 | 4% | 3 | 0.10 | 0.952 |

Note: Percentage and N or mean and SD are reported. Test values are Chi-squared or F-value.

^a^ INOCA group is significantly different from the group(s) with ^a^ using a post-hoc Bonferroni corrected z-test

## Supplemental table S3 Health status, well-being, and psychological distress in men, stratified by cardiac groups

|  | Inconclusive | | INOCA | | CAD obstructive | | Test-value | p-value |
| --- | --- | --- | --- | --- | --- | --- | --- | --- |
| ***Health status and Wellbeing*** |  |  |  |  |  |  |  |  |
| Modified SAQ |  |  |  |  |  |  |  |  |
| Chest pain past month | 63% | 35 | 80% | 16 | 73% | 61 | 2.73 | 0.256 |
| Physical limitation | 55.77 | 20.11 | 54.81 | 24.69 | 54.33 | 22.43 | 0.07 | 0.935 |
| Angina frequency | 79.81 | 20.60 | 77.37 | 16.95 | 77.28 | 23.45 | 0.23 | 0.792 |
| Quality of life | 63.94 | 24.59 | 66.45 | 23.95 | 59.82 | 25.30 | 0.78 | 0.459 |
| Mental Health Continuum |  |  |  |  |  |  |  |  |
| Emotional wellbeing | 3.54 | 1.23 | 3.27 | 1.14 | 3.57 | 1.15 | 0.45 | 0.639 |
| Social wellbeing | 2.64 | 1.10 | 2.86 | 0.95 | 2.68 | 1.21 | 0.24 | 0.783 |
| Psychological wellbeing | 3.34 | 1.01 | 3.16 | 0.97 | 3.36 | 1.00 | 0.30 | 0.740 |
| Wellbeing total | 3.13 | 0.95 | 3.07 | 0.88 | 3.18 | 0.95 | 0.11 | 0.896 |
| Fatigue [FAS10] | 23.40 | 8.41 | 23.50 | 7.93 | 24.11 | 7.27 | 0.15 | 0.859 |
| ***Pychological factors*** |  |  |  |  |  |  |  |  |
| Depressive symptoms [PHQ9] | 5.89 | 6.01 | 5.94 | 6.14 | 5.90 | 5.10 | 0.00 | 0.999 |
| Moderate depression [≥10] | 22% | 12 | 17% | 3 | 22% | 18 | 0.26 | 0.878 |
| Anxiety [GAD7] | 6.25 | 6.20 | 4.94 | 3.63 | 5.13 | 5.45 | 0.73 | 0.484 |
| Moderate anxiety [≥10] | 23% | 12 | 12% | 2 | 15% | 12 | 1.79 | 0.408 |
| Perceived Stress [PSS] | 14.06 | 7.60 | 14.00 | 7.96 | 14.43 | 7.48 | 0.05 | 0.951 |
| Type D personality | 27% | 15 | 37% | 7 | 34% | 28 | 0.89 | 0.642 |
| Negative affectivity [DS14NA] | 10.02 | 6.75 | 9.05 | 6.31 | 9.28 | 6.04 | 0.28 | 0.755 |
| Social Inhibition [DS14SI] | 9.47 | 5.72 | 9.27 | 7.17 | 9.45 | 6.14 | 0.01 | 0.992 |

*Note*: Percentage and N or mean and SD are reported. Test values are chi-squared or F-value.

## Results secondary analysis for INOCA patients stratified for sex

In Supplemental Tables S4 and S5, sex differences are reported for patients with INOCA (which for women included patients with CMD). In total 32% (N=68/213) of women and 13% (N=20/160) of men were classified as having INOCA. Women with INOCA have a higher prevalence of cardiac family history and hypertension, but are less often smokers, less often used any alcohol, less often reported diabetes, and less often used ACE/ARB, beta blockers, and diabetes medication compared to men.

## Supplemental Table S4. INOCA patients’ characteristics stratified by sex

|  | Women (n= 68) | | Men (n= 20) | |  |  |
| --- | --- | --- | --- | --- | --- | --- |
|  | Mean or % | SD or n | Mean or % | SD or n | Test-value | p-value |
| Age [years] | 62.74 | 9.54 | 66.30 | 11.98 | 1.91 | .170 |
| ***Sociodemographic factors*** |  |  |  |  |  |  |
| Having a partner | 90% | 60 | 89% | 17 | 0.00 | .992 |
| College education or higher | 26% | 17 | 32% | 6 | 0.22 | .641 |
| ***Lifestyle factors*** |  |  |  |  |  |  |
| BMI [kg/m2] | 27.04 | 4.93 | 29.05 | 2.86 | 2.71 | .104 |
| Obesity [BMI>=30] | 25% | 16 | 39% | 7 | 1.43 | .231 |
| Current Smoker | 3% | 2 | 25% | 5 | 10.27 | **.001** |
| Any alcohol use | 51% | 34 | 79% | 15 | 4.80 | **.028** |
| Being physically active | 69% | 46 | 61% | 11 | 0.37 | .545 |
| ***Cardiovascular risk factors*** |  |  |  |  |  |  |
| Family history of heart disease | 70% | 47 | 17% | 3 | 16.76 | **<.001** |
| Hypertension | 73% | 48 | 47% | 9 | 4.30 | **.038** |
| Hypercholesterolemia | 65% | 44 | 75% | 15 | 0.74 | .389 |
| Diabetes | 18% | 12 | 45% | 9 | 6.36 | **.012** |
| ***Comorbid conditions*** |  |  |  |  |  |  |
| Migraine | 22% | 15 | 15% | 3 | 0.47 | .491 |
| Fibromyalgia | 13% | 9 | 0% | 0 | 2.95 | .086 |
| Thyroid condition | 15% | 10 | 10% | 2 | 0.29 | .590 |
| Chronic Fatigue Syndrome | 16% | 10 | 17% | 3 | 0.01 | .915 |
| Allergic condition | 23% | 15 | 17% | 3 | 0.38 | .540 |
| Chronic pain | 36% | 23 | 39% | 7 | 0.05 | .818 |
| ***Medication use*** |  |  |  |  |  |  |
| *Cardiac medication* |  |  |  |  |  |  |
| Antithrombotics [B01A] | 59% | 40 | 80% | 16 | 3.00 | .084 |
| Cholesterol lowering [C10A] | 49% | 33 | 70% | 14 | 2.86 | .091 |
| Diuretics [C03] | 22% | 15 | 10% | 2 | 1.44 | .230 |
| ACE/ARB inhibitors [C09] | 28% | 19 | 55% | 11 | 5.04 | **.025** |
| Beta blockers [C07] | 26% | 18 | 80% | 16 | 18.68 | **<.001** |
| Calcium inhibitor [C08] | 50% | 34 | 35% | 7 | 1.40 | .237 |
| Nitrates [C01DA/DX] | 38% | 26 | 40% | 8 | 0.02 | .887 |
| *Other medication* |  |  |  |  |  |  |
| Antidepressants [N06AB/AA/AX] | 15% | 10 | 10% | 2 | 0.29 | .590 |
| Benzodiazepines [N05B/N05C] | 13% | 9 | 5% | 1 | 1.04 | .308 |
| COPD medication [A07/ R03] | 10% | 7 | 5% | 1 | 0.52 | .469 |
| Diabetes medication [A10A/ A10B] | 10% | 7 | 30% | 6 | 4.77 | **.029** |
| Gastric medication [A02] | 41% | 28 | 50% | 10 | 0.49 | .484 |
| Thyroid medication [H03AA01] | 12% | 8 | 5% | 1 | 0.77 | .380 |

Note: Percentage and N or mean and SD are reported. Test values are Chi-squared or F-value.

## Supplemental table S5 Health status, well-being, and psychological distress, stratified by sex

|  | Women | | Men | | Test-value | p-value |
| --- | --- | --- | --- | --- | --- | --- |
| ***Health status and Wellbeing*** |  |  |  |  |  |  |
| Modified SAQ |  |  |  |  |  |  |
| Chest pain past month | 73% | 49 | 80% | 16 | 0.38 | .535 |
| Physical limitation | 60.00 | 19.84 | 54.81 | 24.69 | 0.85 | .359 |
| Angina frequency | 80.76 | 18.75 | 77.37 | 16.95 | 0.50 | .481 |
| Quality of life | 64.81 | 26.51 | 66.45 | 23.95 | 0.06 | .809 |
| Mental health continuum |  |  |  |  |  |  |
| Emotional wellbeing | 3.45 | 0.91 | 3.54 | 1.23 | 0.34 | .582 |
| Social wellbeing | 2.60 | 0.93 | 2.64 | 1.10 | 0.85 | .381 |
| Psychological wellbeing | 3.25 | 1.03 | 3.34 | 1.01 | 0.22 | .642 |
| Wellbeing total score | 3.06 | 0.84 | 3.13 | 0.95 | 0.01 | .914 |
| Fatigue [FAS10] | 24.38 | 7.06 | 23.50 | 7.93 | 0.21 | .650 |
| ***Psychological distress*** |  |  |  |  |  |  |
| Depressive symptoms [PHQ9] | 6.20 | 5.31 | 5.94 | 6.14 | 0.03 | .863 |
| Moderate/severe depression [≥10] | 21% | 14 | 17% | 3 | 0.18 | .670 |
| Anxiety [GAD7] | 6.01 | 5.56 | 4.94 | 3.63 | 0.56 | .457 |
| Moderate/severe anxiety [≥10] | 29% | 19 | 12% | 2 | 2.07 | .150 |
| Perceived Stress [PSS] | 15.80 | 7.61 | 14.00 | 7.96 | 0.78 | .380 |
| Type D personality | 28% | 19 | 37% | 7 | 0.51 | .477 |
| Negative affectivity [DS14-NA] | 11.06 | 5.78 | 9.05 | 6.31 | 1.71 | .195 |
| Social Inhibition [DS14-SI] | 7.52 | 4.86 | 9.27 | 7.17 | 1.53 | .219 |

*Note*: Percentage and N or mean and SD are reported. Test values are Chi-squared or F-value.

1. Kunadian V, Chieffo A, Camici PG, et al. An EAPCI Expert Consensus Document on Ischaemia with Non-Obstructive Coronary Arteries in Collaboration with European Society of CardiologyWorking Group on Coronary Pathophysiology & Microcirculation Endorsed by Coronary Vasomotor Disorders International S. *Eur Heart J*. 2020;44(February):1-21. doi:10.1093/eurheartj/ehaa503 [↑](#footnote-ref-1)
